# Pathology, Practical Medicine, and Therapeutics

**Published:** 1838-01

**Authors:** 


### PATHOLOGY, PRACTICAL MEDICINE, AND THERAPEUTICS.

### *Report of St. John's Fever and Lock Hospitals, Limerick.*
By W. J. Geary, M.D.

In our last Number, (p. 536) we gave a brief notice of this Report, which is now concluded. It is, as we stated, a very valuable document, and is highly creditable to the good sense and zeal of the author. The following extracts are both interesting and important.

I. *Illustrations of the Causes of Fever.*

1. *Suddenness of Contagion.* (Dr. Geary's own case.) "After walking a good deal during the day, the weather being unusually warm, I visited a child, residing in a miserable, ill-ventilated cabin, who was represented to have been ill for several days before. The room in which she lay was very confined, and, having no window, I had her brought to the door of the outer apartment, when she was found to be attacked with maculated typhus. Immediately after examining the case, I was seized with nausea, vertigo, and a general chill. On returning home, I was unable to take my dinner, and, on returning to my room from the table, I stated the circumstances, and my belief that I had caught fever. The disease set in that night, and so urgently did the acute symptoms supervene, that the head was leeched and blistered the next morning. The attack lasted for twenty-one days; and, among other distressing symptoms, singultus continued for seventy-two hours."

2. *Fever from Putrid Effluvia.* "We have seen a very decisive instance of this kind, arising from the effluvia of a foul and ill-conditioned wound of the head, succeeding an extensive fracture of the right parietal bone, with laceration of the dura mater. It occurred in 1819, to my father, while attending an inquest on a young man, who died from the injury. Having sat in the draft, he perceived the 'scent from the wound' intolerably offensive, and felt that he had then inhaled the poisonous dose. In the course of the day he became more sensible of his situation; on the next evening he was obliged to yield, and thenceforward continued in severe and dangerous fever for many days."

3. *Fever from Camphor.* "Miss ——, a young and healthy lady, about twenty years of age, walking by a chemist's shop, was suddenly overpowered by the odour of camphor as she passed the door. She turned to her sister, and exclaimed, 'Oh, did you smell the camphor?—I am sure to have a fever now!' Her predictions were too truly verified; she was attacked with the disease that night, and had a serious illness of several days' duration. This case is the more remarkable as one of the lady's sisters had fever some years before, which was attributable to the 'smell of camphor!'"

II. *Influence of Age.*

"*Table shewing the Number admitted at stated Ages of five Years; their Relation and average Mortality per Cent., from 6th January,* 1836, *to* 5*th January,* 1837.

| Ages, | 5 | 10 | 15 | 20 | 25 | 30 | 35 | 40 | 45 | 50 | 55 | 60 | 65 | 70 | Over | Tot. |
|---|---|---|---|---|---|---|---|---|---|---|---|---|---|---|---|---|
| Admitted, | 81 | 489 | 762 | 701 | 362 | 304 | 100 | 203 | 70 | 82 | 23 | 36 | 2 | 10 | 2 | 3227 |
| Died, | 2 | 13 | 18 | 37 | 22 | 27 | 12 | 45 | 13 | 22 | 5 | 12 | 1 | 5 | 1 | 235 |
| Average Mortality per cent. | 2¼ | 2½ | 2¼ | 5¼ | 6 | 8¾ | 12 | 22¼ | 18½ | 27 | 21½ | 33¼ | 50 | 50 | 50 | 7¼ |

"By comparing the number of admissions and of deaths for successive terms of twenty years, it will be found that the susceptibility to fever diminishes, while the proportionate fatality augments, with the increase of age:—viz. for the first term, in 2033 admissions, the deaths were 3½ per cent., while, for two succeeding periods of equal duration, the mortality reached 11 and 25 per cent. respectively; the admissions being 969 and 211. Of the entire treated for the year, full two-thirds were under twenty years of age; and only one-third of the mortality occurred within that term. These deductions shew satisfactorily the influence which age has both on the extent and fatality of fever cases."

III. *Influence of Sex.*

"The influence of sex appears in favour of the males, one-third less having been attacked than of the females. The greater delicacy of the latter, as well as the disproportionate amount of labour they undergo, slenderly clothed, and bare-footed, seem to be the principal cause of their greater susceptibility to fever. As regards the mortality, there does not appear to be any remarkable difference, the proportion being 1 in 13⅓ of females, and 1 in 14 of males; though that for six years, from 1814 to 1820, gave the proportion of females as 22 to 15; a relative average which commonly exists."

### IV. *Influence of the Period of commencing Treatment on the Mortality.*

"Among the assignable causes of mortality, too much importance cannot be attached to the period of disease at which the sick are brought to hospital. It seems a well-established point, that the chances of recovery are in the exact ratio of the period of illness at which the sick come under treatment. . . . . The beneficial result of early treatment is clearly represented in the following table.

| Day of illness when admitted, | 2 | 3 | 4 | 5 | 6 | 7 | 8 | 9 | 10 | 11 | 12 | 13 | 14 | Over | Total. |
|---|---|---|---|---|---|---|---|---|---|---|---|---|---|---|---|
| No.ofCases | 84 | 319 | 484 | 538 | 472 | 289 | 479 | 170 | 131 | 28 | 41 | 16 | 67 | 88 | 3206 |
| Deaths, | 3 | 10 | 22 | 28 | 33 | 30 | 36 | 15 | 21 | 2 | 5 | 1 | 5 | 24 | 235 |
| Mortality per cent. | $3\frac{1}{4}$ | $3\frac{1}{8}$ | $4\frac{1}{2}$ | $5\frac{1}{8}$ | 7 | $10\frac{1}{4}$ | $7\frac{1}{2}$ | 9 | 16 | 7 | $12\frac{1}{8}$ | $6\frac{1}{4}$ | $7\frac{1}{2}$ | $27\frac{1}{4}$ | $7\frac{1}{4}$ |

*Dublin Journal.* *September*, 1837.

---

### *On the Inadequacy of the Valves of the Aorta.* By Wm. Henderson, m.d.

In this paper the author reviews the state of our knowledge respecting the diagnosis of this affection, as successively improved by the observations of Drs. Corrigan, Hope, Guyot, Charcelay, M'Adam, and Watson. In addition to the signs already announced, Dr. Henderson gives a new one from his own experience; viz. "a greatly increased interval between the systole of the heart and the pulse of the remote arteries, such as the radial." . . . "The increased interval," says Dr. H. "was so great that the radial pulse exactly alternated with the systole of the ventricles, or occurred in the middle of the interval between the successive pulses of the heart. I have examined many persons affected with disease of the heart in different forms, and found this peculiarity of the pulse in none, except those in whom there were signs of defective action of the aortic valves."

In the following extract, Dr. H. "sums up what appears to be warranted regarding the more prominent signs" of this affection.

"1st. Visible pulsation, or remarkable leaping of the arteries of the head and upper extremities, is a very common attendant on the disease; yet not worthy of being considered a pathognomic sign, because it is not always present in a degree of peculiar significance, may result merely from a greatly enlarged left ventricle, (to which, indeed, M. Guyot attributes it in patency of the valves,) and may be caused by continued nervous excitement, (Corrigan;) nor is its true cause revealed by,

"2dly. Bruit de soufflet and fremissement, accompanying the diastole of the arteries, as maintained by Dr. Corrigan; for the former of these may result from mere irritability, (Hope) and the latter is often absent altogether in cases of patency. These phenomena may also result from disproportion between an enlarged ventricle and the orifice of the aorta, and from vegetations on the valves.

"3dly. A bellows-murmur, in place of the usual second sound, most audible along the track of the aorta and its primary branches, is an indication of patency of the aortic valves, except in those rare cases of aneurism in the course of the sub-sternal aorta which have the power of originating two murmurs. The musical or cooing note, in place of the second sound, has hitherto been found to originate only in cases of patency of these valves. The morbid murmur during the diastole of the heart, whether a bellows-sound or a sonorous note, may, contrary to the opinion of Dr. Hope, be very loud in cases of incompetency of the valves. In some cases, however, there are no such sounds.

"4thly. The occurrence of a preternatural interval between the period of the heart's contraction and of the remote arterial pulse, promises to prove a valuable sign of inadequacy of the aortic valves. Should it prove, on further enquiry, to be constant, it will remove the difficulties in the differential diagnosis of patency.

"5thly. The usual full pulse and tortuous state of the arteries are natural consequences of the great volume of the left ventricle, which, since the time of Haller, has been known to result from insufficiency of the semilunar valves."

*Edinburgh Journal. Oct.* 1837.

---

*Effects of deficient Ossification of the Cranium.* By JOHN GRANTHAM, Esq , Surgeon, Crayford.

OUR readers will remember the important observations first made by the late Sir Gilbert Blane, of the necessity of proper support from the cranium to enable the brain to execute its functions; and the practice founded thereon of bandaging the head in certain cases of hydrocephalus. In the present communication, Mr. Grantham adduces several cases to shew that epilepsy, cerebral congestion, hydrocephalus, &c., are often the consequences of imperfect ossification of the scull in infants, and are relieved by bandaging the head. The titles of the first three cases will shew the nature of the author's views and practice:

1. Epileptic fits—open sutures—roller to the head—recovery. 2. Epileptic fits—temporary improvement from the application of roller—death. 3. Restlessness and general indisposition—bandage to the head—recovery.

*Med. Gazette. Sept.* 1, 1837.

---

*On Aortitis, as one of the Causes of Angina Pectoris; with Observations on its Nature and Treatment.* By D. J. CORRIGAN, M.D., Physician to the Jervis-street Hospital, Dublin.

THIS is a valuable contribution to the pathology of the diseases of the heart and great vessels, and throws some valuable light on that complex and occasionally obscure affection, angina pectoris. The object of the paper is, "1st, to shew that *inflammation of the lining membrane of the mouth of the aorta* is capable of producing the group of symptoms to which we give the name of angina pectoris, and is therefore entitled to a place in the list of the causes of that formidable affection; 2dly, to trace the pathology and treatment of aortitis." With these views, he relates three series of cases, first, "cases in which the patient died, while the disease was acute or recent; then those cases of longer duration, which exhibit the alterations of structure produced by the disease, when uncontrolled in its progress; and, lastly, those cases in which, from analogy with the symptoms of the former cases, there was every reason to suppose the existence of the disease, and in which treatment founded on the supposition was attended with success."

The first class contains two dissections, from Portal and Bouillaud, of patients dying suddenly from attacks of suffocative dyspnœa and palpitation, in whose bodies dissection shewed evident signs of recent inflammation at the origin of the aorta; and two cases observed by the author. The following is one of them:

Glynn, æt. 44, "had been complaining, for three months previously, of debility and cough, accompanied with attacks of dyspnœa, attacking him when walking or working, and obliging him to stop frequently to sit down. He also suffered acutely from sensations as if of tearing asunder in his chest. After exercise, he suffered from palpitation.

"Physical examination of the chest gave extensive dulness over the præcordial region, with tumultuous and indistinct action of the heart, and puerile respiration in the lungs, but no other sign of disease On the 24th, five days after admission, head symptoms set in,—viz. dry retching, followed by delirium and stupor; and he died on the 26th.

"*P. M.* There was some fluid in the pericardium; the whole heart was enlarged, the left ventricle and auricle particularly so. The lining membrane of the aorta, just above the valves, was of a vivid red colour, and was protruded considerably beyond the natural level by an effusion of red and (apparently) organized lymph, which lay behind it, effused between it and the fibrous coat of the vessel

This vividly red and swollen portion of the vessel contrasted strongly with the pale and polished surface of the artery a little further on."

There is only one case of the second class, which exhibited during life the symptoms of disease of the heart and angina pectoris. "On dissection, the semilunar valves of the aorta were found perforated and cartilaginous, and the lining membrane of the aorta, from the mouth of the vessel to its arch, contained underneath it innumerable atheromatous depositions."

The third class contains two cases, supposed to be examples of inflammation of the aorta, and cured by treatment administered with this view. The following is one of them:

"In February, 1835, Mr. D. passed through a very severe attack of acute rheumatism, and in the course of it was frequently seized by what were supposed to be fits of spasmodic dyspnœa. I saw him in his convalescence. He was suffering little from articular pains, but there was very strong action of the heart, with indistinct bruit de soufflet; and exercise brought on severe palpitations. I warned him of his danger, but in vain. He was so impressed with the conviction of his heart symptoms being nervous, that no persuasion could induce him to submit to treatment conducted on any other supposition. In eighteen months afterwards he came to town to consult me: his lips were livid and his feet œdematous, and he was suffering from severe and frequent paroxysms of dyspnœa. He dreaded to lie down, and the fits of palpitation and dyspnœa were brought on by any exercise, but more particularly by attempting to walk up an ascent. The abdominal organs were sound. The chest sounded well on percussion; and the respiration was in every part natural, or somewhat puerile. The pulsation of the heart was felt over a large space with strong impulse, and there was very slight bruit de soufflet in the præcordial region; pulse was about eighty-five, and small. These were the symptoms on the 4th of August, 1836. Leeches were applied over the region of the heart, and ten grains of hyd. c. magnesia given three times a day; while abstinence from wine and from stimulant antispasmodics was strictly enjoined. A seton was inserted before the left mamma.

"In four days the mouth was made sore, and the change in his state was as gratifying as it was rapid. The breathing became easy, the fits of dyspnœa ceased, and he slept without disturbance and without dread in the recumbent posture.

"On the 22d, I again made his mouth sore. He then returned home, and has never had any return of his former distressing symptoms. While these sheets are going through the press, (October, 1837,) I have again had an opportunity of seeing him. He is in perfectly good health, after having undergone the exciting and arduous exertions attendant on taking an active part in two of the most violently contested elections in the kingdom."

The other case was treated in a similar manner.

The following conclusions are deduced by Dr. Corrigan from the review of the premises:

"1st. That, in some cases of what are called angina pectoris, the paroxysms of dyspnœa, anxiety, mental distress, &c., constituting a fit of angina pectoris, and often supposed to be merely nervous, are really the symptoms of aortitis, or inflammation of the mouth of the aorta.

"2d. That the treatment, in such cases, is the adoption of local bleeding, counter-irritation, and the exhibition of mercury, which experience has taught us are the means best calculated to prevent the effusion or cause the absorption of lymph.

"3d. The pathology of the disease, which these cases have enabled us to trace, encourages us also to put into requisition our treatment, and to persevere in it after even a considerable lapse of time has passed by, in instances too where, without this knowledge, we should have looked upon the case in despair, from the belief that irremediable organic disease had been established."

These observations are highly important in a practical point of view, and deserve attention. We believe that many cases of affections of the heart and large vessels are lost, from the false conviction of their hopelessness entertained by practitioners. On the whole, we think Dr. Corrigan has made out his case; although the coexistence of disease of the heart, along with that of the aorta, in some of his cases, renders the conclusion, that the aortitis was the cause of the angina, not quite logical. *Dublin Journal. Nov.* 1837.

---

*On the Remedial Powers of the Aconitum Napellus in Headach.*
By W. C. RADLEY, Esq., Surgeon, Newton Abbot.

MR. RADLEY was induced to try this remedy, many years since, at the recommendation of the late Dr. Turton, the botanist; and found it most efficacious. The cases in which the aconite was successful (for it was not always so,) were those of idiopathic nervous headach, not dependent on other causes; and it acted better on the delicate and nervous than on the sanguine and robust. Six cases are related, in four of which it produced almost an immediate cure, and in one constant relief in the attacks. "The form which I have used," says Mr. R. "is the simple extract made from the inspissated juice expressed from the bruised leaves of fresh-gathered plants, in the latter end of May, just before the time of flowering, poured into shallow vessels of earthenware, and allowed to evaporate slowly in the shade in warm weather. Its consistence is tough, colour nearly black; it keeps well, and a few grains, the last of about a pound made nineteen years ago, are as good and as efficacious now as they then were."

This extract was administered in the form of pill, in doses varying from one grain to two and a half grains; the smaller dose being preferred. The pill was repeated according to its effects: sometimes a single dose seems to have effected a cure.

*Lancet. Sept.* 23, 1837.

---

*On the Use of Tartar Emetic and Opium in Spasmodic Affections.*
By R. G. ACKERLEY, Esq., Surgeon, Liverpool.

THE author deprecates, and with much propriety, the habitual employment by practitioners, of that class of remedies usually called antispasmodics, but which are in fact powerful stimulants, in cases of spasm. Mr. A. thinks that we should rather look to remedies "possessing power of relieving the excited state of the brain and nervous system, and thus allaying the tonic muscular contractions which characterize this class of diseases." He fulfils his indication as follows:

"The remedy which I propose, (and it is one which I have used with the greatest success for the last three years,) is the tartar emetic, in combination with opium in some of its forms. The sedative effects of the opium are powerfully increased by uniting with it the antimonial salt, while its narcotic properties are diminished. The manner in which I prescribe it is the following:—Three or four grains of tartar emetic, with two drachms of laudanum, or one of Battley's sedative liquor of opium, and two ounces of water. A tea-spoonful of this mixture to be given every fifteen minutes, until relief is obtained, and afterwards every hour, until all symptoms of the affection have disappeared. Its first effect is generally to produce nausea, or even vomiting. The latter I encourage, where, as is frequently the case, I have every reason to suspect that the spasm proceeds from improper or undigested food in the stomach, acting as a source of irritation. After this, the medicine is generally retained; *tolerance*, as Rasori describes it, being established, and the spasms speedily subside. It may be given in spasm of the stomach, diaphragm, spasmodic asthma, and during the paroxysm of hysteria, with the most beneficial results. I have administered it in such cases in the advanced stage of pregnancy; nor do I consider an irritable state of the stomach, with vomiting, any objection to the use of it, the sickness generally subsiding after the second or third dose. Should the symptoms be very urgent, if the case will in other respects allow of it, I do not hesitate to bleed, as a most powerful auxiliary; but phlebotomy may in general be dispensed with. Enemata are also at times beneficial, particularly where the attack is accompanied by constipation, though the tartar emetic sometimes produces evacuations from the bowels, and renders their administration unnecessary."

*Med. Gazette. October* 7, 1837.

*Observations on the Nature of Neuralgia, and on the Principles according to which the Treatment ought to be conducted.* By JONATHAN OSBORNE, M.D., Dublin.

THIS paper contains some interesting facts and remarks, but we doubt if the author's views are so original as he supposes. He says,

"After a due consideration of the recorded facts, and a comparison of them with the cases under my own observation, I have for some time past arrived at the conclusion, that the nature of neuralgia is altogether different from any of the opinions respecting it now mentioned; and that it is nothing else than pain arising from paralysis of the nerve sufficient to alter its mode of sensation, but not so complete as to obliterate it."

In proof of the accuracy of this opinion, he adduces many facts and arguments, the principal of which we will give in an abridged form:

"1. Pressure on a sound nerve produces a kind of neuralgia.

2. The affections usually accompanying neuralgia are of a paralytic character.

3. The *adjuvantia* and *lædentia* prove the disease not to be one of excitement. Sedatives rarely give relief, while tonics and stimulants often do.

4. Inflammation of a nerve or its sheath is marked by different symptoms from those of neuralgia.

5. The recorded cases of neuralgia from supposed irritation of a foreign body originated from pressure on the nerve, producing paralysis.

6. In neuralgia from indigestion, &c., the accompanying symptoms point to paralysis, rather than any other state.

7. The pain accompanying various organic diseases are really neuralgic, and arise from pressure produced by the organic disease. The intercostal and other pains commonly attributed to spinal irritation are rather to be viewed as the consequence of vascular congestion, pressing on the nerves at their origin, and thus causing imperfect paralysis."

"11th. What appears to me, however, the strongest and most decisive argument that neuralgia is a torpid state of a nerve, is derived from the effect of acupuncture, which cannot be conceived to act in any way than as an irritant."

In illustration of the truth of his theory, and of the efficacy of acupuncture, he gives the following interesting case.

"Mr. M., aged thirty-six, during the last two years the left side, but during four years previously, the right side, was the chief seat of pain. His sleep was only obtained during short intermissions of pain, which, although never entirely absent when awake, was yet variable, and at times increased to the utmost degree of violence. The mouth was slightly drawn upwards to the left side; the motion of the opposite side impeded. The tongue slightly protruded towards the left; the right eye directed inwards from the axis of vision. Within about six months from the commencement of his illness, he gradually lost the sight of the right eye, and about the same time the hearing of the right ear. His sight had been recovered, but in the right ear hearing remained defective, with the sensation of a buzzing noise in it." . . . "I commenced the local treatment by rubbing over the affected part the extract of belladonna, which was kept constantly applied. Having on former occasions seen much relief from this in slight cases of facial neuralgia, it was desirable to see what it could effect in this case of six years' standing. He experienced no appreciable action beyond the prickling sensation it causes on the skin, and the dilatation of the pupil. Acupuncture was then practised behind the ear, and about the zygomatic arch. It was followed by a remission of pain greater than he had hitherto ever experienced. Next day the pain behind the ear again began to be felt: the needles were applied there in greater number. On the following day the pains were reported as fugacious, and rather resembling an apprehension of its recurrence than pain actually present. On several following days he continued in this state, but his nights continued sleepless, although free from pain. Considering that his want of sleep was now kept up principally by the habit of lying awake, I gave him acetate of morphine, in doses of one-sixth of a grain, in solution, every

3

two hours, commencing in the evening. This proving inadequate, he got twenty drops of the black drop. Sleep was at once restored. His general health, which had suffered much, began rapidly to amend. The pulse, previously 116 and hard, fell to 84, and became soft. About four nights afterwards the pain recommenced behind the ear, and further back than when acupuncture had been applied. Needles were applied to that part, with the effect of completely dislodging the pain."

The following is Dr. O.'s account of his mode of using acupuncture.

"Acupuncture being, according to my opinion, indicated exclusively in neuralgia, and being in it a most powerful remedy, which rouses the nerve from incomplete to complete sensation; its efficacy is greatest when the nerves themselves are transfixed in the greatest number. In tic douloureux of the face, as the nerves of that part are spread over a greater extent of surface than in the fleshy parts of the lower extremities, which are the seat of sciatica, it is evident that the chance of nerves being transfixed is much less; and hence, I think, acupuncture will in these cases require to be more frequently repeated. The needles which I use in facial neuralgia are less than one-half inch in length, and are generally the pointed ends of well-tempered needles broken off. They must also be inserted in greater number."

*Dublin Journal. November*, 1837.

---

*On artificial Respiration in the Asphyxia of Still-born Children, and during Convulsive Fits.* By LAWSON CAPE, M.D.

THE practice here recommended has been long in use in the first class of cases. Mr. Terry, of Northampton, seems to have the credit of having first enforced the great importance of long-continued perseverance in it, even in apparently hopeless cases.

"In one instance related by him, resuscitation took place after two hours and a half; and in the other, after one hour and three-quarters: in the latter instance the child being completely restored. I have met with several instances where I have been equally successful, when the action of the heart was not in the least perceptible, and where, for a very considerable time, all appearance of vitality was absent, and yet, after artificial respiration had been kept up for a time in which perseverance seemed hopeless, the action of the heart began to be distinguished, the livid aspect gave place to the colours of health, and the lungs at length were called into action."

Dr. Cape strongly recommends the same means in the asphyxia frequently met with in the convulsive fits of children, and gives an interesting case illustrative of its effects. The infant was five days old, and had a succession of violent convulsions for thirteen or fourteen hours, on the average of a fit each hour.

"The pressure produced upon the brain was such that respiration was entirely suspended during the greater part of the fits, and even the action of the heart could not be felt for more than ten minutes in the third and twelfth fits, and the child lay to all appearance dead. It was at such times that I proceeded to restore the suspended functions by artificially inflating the lungs, in the manner described by Mr. Terry: namely, by breathing into the mouth of the infant from my own, closing the nostrils, and compressing the thorax after each inflation; observing the natural periods of frequency as much as possible. I am quite convinced the child would have been lost, had it not been for the artificial aid thus afforded to nature in the severe struggle, till the offending matter was expelled. By means of the artificial respiration, the colour (especially of the face and lips) turned from purple to red, but still there was no breathing, till a convulsive gasp announced the termination of the fit."

In this case the child recovered perfectly.

*Med. Gazette. October* 7, 1837.

### *Disease of the Spino-occipital Articulation, with the appearances on Dissection.* By T. R. Blackley, M.R.C.S. &c.

This is a very valuable case in relation to headach and diseases of the spine, because it affords an instance of what may be termed physical pathology; a rare event in regard to these affections. It was not necessary to prove the intermitting character of *symptoms* depending on a fixed organic cause; but it is a good example of this common fact.

"Mary King, aged eight years, was admitted March 17th, 1837, into the Hospital for Children, Portobello, for violent headaches, to which she had been very subject for the last two months; these pains commenced suddenly on her return in the evening from the funeral of her sister, to whom she was much attached.

"Her countenance was peculiarly expressive of caution, and was florid, and full, if not bloated; the chin was advanced preternaturally beyond the chest, the mouth slightly opened, and she kept the arms parted from the sides as if to poise herself. On looking laterally, she strained her eyes in the direction of the object, and failing in this, turned her entire body for the purpose. The effect produced when she attempted to observe anything placed near her feet was yet more remarkable; for this purpose she generally put her hand to her forehead, as if fearful of undue weight in the head, and bent her body, thus avoiding the least motion between the first and second vertebræ. In getting up from bed also, or in lying down, she invariably supported the head with her hand.

"The headach was not permanent, sometimes she was free from pain for a couple of days, especially while using the warm bath, and on those occasions exhibited a most cheerful disposition; as the disease advanced, however, the intervals of rest were fewer and of shorter duration; the use of calomel seemed to afford considerable ease, until it slightly affected the mouth on the fifth day, from which time her sufferings appeared rather to be aggravated; leeching and blistering, which had been tried at the commencement of my treatment, to the back of her neck, were perfectly useless, nor was the insertion of a seton in the same situation attended by any happier result. The sufferings of this poor little creature were really most painful to witness, and at times amounted to agony; on these occasions she always wished to have a bandage bound tightly round her forehead, or requested the nurse to squeeze her head between her hands, but the most effectual remedy was the tincture of opium in small quantities, repeated when necessary. Of late she referred the headach more to the left than the right side; the pain on several occasions left the head, and traversed the spine to the sacrum, from whence it returned to its original seat; she also complained occasionally of pain in her stomach, but this was always relieved by the application of a jar of hot water to the part; the bowels were regular throughout.

"She expired without a struggle on the 29th June. Owing to the objections entertained by her friends to have the body examined, I was obliged to content myself with the removal of the diseased portions, namely, the cervical portion of the spine, and the portion of occipital bone in connexion with it; and with my friend Dr. Houston made a minute examination, the result of which was as follows:

"The articulating surfaces between the first and second vertebræ, and between the condyles of the atlas and occipital bone, were diseased, the cartilages eroded, the denuded bones gritty, and the capsules thickened and coated with lymph. The capsules of the loose articulations between the oblique processes of the atlas and dentata were particularly enlarged, and protruded forwards under the deep muscles of the spine, forming three distinct abscesses pressing against the pharynx; that on the right side had made its way downwards for more than an inch between the bones and muscles. The odontoid process was completely detached on all sides, and stripped of cartilage and synovial membrane, it was immersed in pus. The perpendicular, oblique, and transverse ligaments had altogether disappeared, and the odontoid process was only separated from the spinal marrow by the thickened sheath of that organ. All the joints enumerated, including those of the odontoid process, appeared to be engaged in one suppurating surface. The fibrous sheath of the medulla oblongata, and commencement of the spinal marrow, were much thickened in texture, and the arachnoid and pia mater to the same extent were red and swollen; but the roots of the nerves were all perfect, and the medullary texture did not exhibit any organic lesion. The latter was perhaps a little softened, but had not undergone any alteration of colour; I say a *little* softened, because, as Dr. Houston remarked, in the healthy state this part of the spinal marrow feels more resisting than any other, and in this instance it yielded most easily to pressure with the finger." *Dublin Journal. Sept.* 1837.

---

*On Acupuncture in Hydrocele and Ascites.* By D. LEWIS, Esq., and T. KING, Esq.

ON a former occasion (*Brit. and For. Med. Rev.* Vol. III. p. 559,) we noticed Mr. Lewis's novel mode of treating hydrocele by acupuncture, and referred to its employment in other cases of circumscribed dropsy. In the present short paper, he records the result of his further experience of the practice, and adds an interesting case of its application to ascites by Mr. King. The following extracts specify both methods:

1. "Although I have seen radical cures take place from the simple operation of a single puncture, yet, as acupuncture is not a radical cure in all cases, I beg to submit to my professional brethren the result of my own experience. My method is to make a single puncture twice a week, for the first few weeks, so as to keep the tunica vaginalis in a collapsed state, and to bring on a healthy instead of a morbid secretion: by so doing, I find that the disposition to secrete water diminishes; and, therefore, if, on examination, I find only a drachm of fluid, I puncture. By pursuing this method, and employing at the same time the following ointment,—℞. Hydriod. Pot. ʒss.; Ung. Cetacei, ℥jss. M. ft. unguent.—twice a day, a radical cure is effected very speedily in recent hydrocele; but, when the hydrocele has been of ancient date, the tunica vaginalis is so thickened that, although the fluid is absorbed, the parts do not appear to diminish, the thickening of the part preventing any evident contraction."

2. The disease shewed itself in July. "Upon deliberation, we were of opinion that some benefit might accrue to him from puncturing the abdomen with a long needle, (of the size used for darning,) so as to give the effused serum an opportunity of oozing from the peritoneal sac into the subcutaneous cellular tissue. The first puncture was made September 2d, on the right side, midway between the umbilicus and spine of the ilium, and was followed, as I withdrew the needle, by the issuing of three or four drops, one by one, of yellow serum. September 3d, another was made, anteriorly to the first. September 4th, there was a marked diminution in the tension. The abdomen was quite altered to the touch; the cellular tissue, which in this region is two inches thick, presenting an œdematous state, in lieu of the tense unyielding condition it was in prior to the operations. A third puncture was made between the other two. September 7th, I made two more punctures. 9th. Amelioration still marked, and the patient's general health is improving. As every operation was followed by some amendment, I made three punctures on the 11th, three on the 16th, three on the 18th, five on the 20th, six on the 21st, six on the 22d, and seven this day. The result may be stated to be a diminution of four inches in the circumference of the abdomen, and an amendment of the patient's general health, such that he is able to walk out."

*Med. Gazette. Oct.* 7, 1837.

In a subsequent communication, dated November 13th, Mr. King informs us that, since the former report, he has continued the same treatment, having made thirty-three additional punctures; and that the patient is, at the time of his writing, almost restored to health, the abdomen being reduced from four feet four inches to three feet four inches in circumference. The following account of the apparent mode of action of this treatment is curious:

"The fluid, with the exception of a small quantity which escapes through the opening in the skin, oozes for three or four hours from the cavity of the peritoneum into the subcutaneous cellular tissue, which it loads and œdematizes. From hence it is gradually absorbed, and chiefly during the next five or six hours; after which the patient voids a good deal of urine, and often perspires pretty freely. This process appears to be continued or repeated more or less completely until the peritoneal orifices of the punctures close. Thus has the patient been relieved of the greater part of the fluid which the peritoneum contained." *Ib. Nov.* 25, 1837.

---

*On the Treatment of Spasmodic Cholera by Sugar of Lead and Opium.*
By R. J. Graves, m.d., of Dublin.

Sceptics as we are of an efficacious remedy being likely to be soon found out for the true and terrible Asiatic cholera, and utterly distrusting the power of *all* heretofore proposed,—most of them with such blind and unphilosophical confidence,—we cannot fail being struck by that here so strongly recommended by a physician of such talent and experience as Dr. Graves. During the prevalence of cholera in Dublin, in 1834, Dr. G., having had occasion to treat some cases of diarrhœa in fever with large doses of acetate of lead, according to Dr. Bardsley's plan, and being struck with its efficacy in such cases, was induced to give it first in dysentery, and then in cholera. The following extracts exhibit Dr. Graves's mode of treatment, and its very striking results:

"I saw a case of cholera, still in the stage of premonitory diarrhœa, or rather just passing from the bowel complaint into the fully formed disease. I tried the acetate of lead boldly, and with the happiest success. Thus encouraged, I applied this new method of treatment in every case to which I was called, and I was employed both night and day in visiting cholera patients, and every hour gave me additional proofs of the efficacy of the remedy. My formula was as follows:

℞. Acetatis Plumbi, ℈j.; Opii, gr. j. M. fiat secundum artem massa, in pilul. xij. dividenda.*

"The premonitory diarrhœa has almost invariably stopped by taking one of these pills, at first every hour, and, as the stools became less frequent, every third or sixth hour, according to circumstances. When the vomiting, spasms, and the state of collapse had begun, it was necessary to give a pill every quarter of an hour: after a couple of hours the effect of the pills became perceptible, in a diminution of the serous evacuations upwards and downwards; then the pills were given only every hour; and as the symptoms yielded they were given less and less frequently, and could in general be laid aside altogether before twenty-four hours. In some it was found necessary to give the acetate of lead in solution, combined with a little vinegar and minute doses of acetate of morphia. Minute doses of opium were useful; anything of large doses hurtful. . . . Many took more than forty grains of the acetate of lead in twenty-four hours: it usually darkened, or even blackened, the alvine discharges, before they ceased altogether. Were I to enumerate all the cases of violent cholera that yielded to this treatment, I would be led into a tedious but not an uninstructive detail. . . .

"After I found out the benefits resulting from the employment of acetate of lead, I no longer desponded when called to cases of cholera, knowing that in the great majority of instances the disease would yield. Of course, there are cases of cholera which admit of no treatment, and which an experienced eye will at once recognize as fatal: they occur generally among the aged or the very young, and are fatal in the course of a few hours, often without any premonitory diarrhœa. But this constitutes no valid objection to the practice; for in what disease do not cases occur which baffle all our efforts?

"I cannot conclude," says Dr. Graves, "without imploring the profession, in every part of the world where cholera prevails, to give my plan of treatment a fair trial; for I feel confident of its efficacy."

*Med. Gazette. October* 14, 1837.

* The most convenient way of making these pills is to add five or six grains of powdered liquorice to the scruple of the acetate of lead, forming a mass by mucilage.

*On the Use of Colchicum in Scarlatina.* By Wm. Tait, Esq., Surgeon.

In our last Volume (pp. 249, 565,) we gave an account of the employment of this remedy by Dr. Lewin in fever. Mr. Tait here informs us, that he had employed it in scarlatina, without any knowledge of Dr. Lewin's practice.

"I administered it only to thirty-five patients, being little more than one-fourth of those for which I prescribed; but these, of course, were of the worst description, being all of the pure inflammatory type. In the most of these, bloodletting, both general and local, was had recourse to; in others, local bleeding only; and I may here remark, that the effects of the colchicum were always most apparent after detraction of blood; but in all the following changes were more or less manifested in a short time before its administration. The pulse was diminished in frequency and force; the palpitation of the heart, which in young subjects was often perceptible to the eye, subsided; the inflammation and pain of the throat were alleviated; and the patient often expressed himself 'much better.' Vomiting was excited in a few cases; but, as this seemed always to be followed by an improvement in a state of the tonsils, and generally abated after the rejection of a quantity of bile, it was never found necessary to interrupt the use of the medicine. The bowels were generally more or less purged, and the improvement was always so sudden and marked, after a free discharge of dark bilious stools, that I always considered my patient out of danger the moment they appeared. In some cases, where bloodletting was not premised, these effects were not so easily produced; two days having elapsed in one case before any change was observable.

"When called to a case of inflammatory scarlatina, it was invariably my practice, after administering a purgative, and bleeding from the arm, or locally by leeches, (as circumstances might require,) to begin with the Vinum Colchici, and continue it till all the inflammatory symptoms were subdued; a blister round the throat being all that was necessary to complete the cure. Almost the only gargle used was a little warm water, and the occasional inhalation of vapour; and with this, and the treatment above detailed, I have the utmost satisfaction in saying that I never saw a tonsil or any part of the mouth ulcerated. The dose of the Vinum Colchici was considerably below that administered by Dr. Lewins, and differed according to the age and strength of the patient. In no case did it exceed twelve or fifteen drops every three or four hours, in a little water, sweetened with syrup, and this only in strong, robust farm-servants: for children of four or six years, I began with three or four drops, and decreased the dose, watching its effects, and stopped whenever these were manifested."

Mr. Tait describes his success as singularly great, both actually and comparatively with that of his brother practitioners in the same epidemic. He only lost one patient out of 126, he says; while others lost one in five or six. We cannot believe that so great a difference as this could result from any difference of treatment. *Lancet. Nov.* 4, 1837.

